# A new interpretable belief rule base model with step-length convergence strategy for aerospace relay health state assessment

**DOI:** 10.1038/s41598-023-41305-z

**Published:** 2023-08-28

**Authors:** Xiuxian Yin, Bing Xu, Laihong Hu, Hongyu Li, Wei He

**Affiliations:** 1 Harbin Normal University, Harbin, 150025 China; 2 Rocket Force University of Engineering, Xi’an, 710025 China

**Keywords:** Aerospace engineering, Computer science

## Abstract

Health state assessment is an important measure to maintain the safety of aerospace relays. Due to the uncertainty within the relay system, the accuracy of the model assessment is challenged. In addition, the opaqueness of the process and incomprehensibility of the results tend to lose trust in the model, especially in high security fields, so it is crucial to maintain the interpretability of the model. Thus, this paper proposes a new interpretable belief rule base model with step-length convergence strategy (IBRB-Sc) for aerospace relay health state assessment. First, general interpretability criteria for BRB are considered, and strategies for maintaining model interpretability are designed. Second, the evidential reasoning (ER) method is used as the inference machine. Then, optimization is performed based on the Interpretable Projection Covariance Matrix Adaptive Evolution Strategy (IP-CMA-ES). Finally, the validity of the model is verified using the JRC-7M aerospace relay as a case study. Comparative experiments show that the proposed model maintains high accuracy and achieves advantages in interpretability.

## Introduction

Maintenance of aerospace systems is an important aspect of ensuring the maintenance of the safety and reliability of astronauts and space equipment^1–3^. One key component of maintenance is the assessment of the health state, which involves detecting and diagnosing faults or anomalies that may affect the performance of the system^4^.

Aerospace relays are critical components in the operation of aircraft systems, such as avionics, flight control, and power distribution^5^. The health state of a relay can be affected by various factors, such as aging, environmental conditions, and mechanical stress. Traditional methods for relay health state assessment rely on manual inspections and tests, which can be time-consuming and costly. Furthermore, manual inspections may not always detect subtle faults or anomalies that could lead to failures. With continued research and development, researchers have developed advanced and sophisticated methods for assessing the health state of complex systems. There are three main assessment methods: models that are data-based, those that are based on knowledge, and those that combine both data and knowledge^6–8^.

Data-based models can provide accurate predictions or classifications without requiring prior knowledge or understanding of the underlying system. They can handle complex, high-dimensional data and can often outperform knowledge-based models in terms of predictive accuracy. In addition, they can learn and adapt to new data. They can continuously improve their performance by adjusting their internal parameters in response to new observations^9^. Guo et al. used a convolutional neural network to identify the health state of bearings, and the network parameters were learned using an error back propagation algorithm and an adaptive moment estimation algorithm. The method improved performance compared to other methods^10^. Liu et al. used a competitive learning-based back-propagation neural network to predict tool condition and tool wear based on three-axis cutting force data and successfully achieved real-time identification and measurement using various network structures^11^. Tamilselvan et al. used deep belief networks for multi-sensor health diagnosis, which involves a hierarchical structure and continuous learning process. The proposed methodology is demonstrated to be effective^12^. Lamsumang et al. used dynamic Bayesian networks to model the degradation of complex engineering systems to assess the health status of quadrotor UAVs online^13^. Wang et al. proposed a Bayesian network fusing hardware health status and data link health status for evaluating the health status of UAVs^14^.

Knowledge-based models are machine learning models where the internal workings and decision-making processes are transparent and easily interpretable by humans. This means that the model's decision-making process is easily explained, and the reasons for a particular prediction or decision can be traced back to specific features or input variables. Zheng et al. used the first-right method, hierarchical analysis method, cloud model, and fuzzy integrated judgment method to evaluate the health status of wind turbines^15^. Li et al. used the coefficient of variation method and the improved fuzzy integrated evaluation method to assess the health status of wind turbines^16^. Arshad et al. used fuzzy logic inference to evaluate the health status of transformer oil-paper insulation system^17^.

However, data-based models can be difficult to interpret^7,8,18^. Their lack of transparency can be a problem in certain applications, such as healthcare, finance, or security complex systems, where interpretability and accountability are important. Knowledge-based models have limited ability to handle complex, nonlinear relationships between input and output variables^7,8^. Due to their simple structure and explicit mathematical formulation, they may not capture the true underlying relationships between variables, especially when the relationships are highly complex and nonlinear.

Therefore, hybrid models that combine knowledge and data are particularly important. Feng et al. proposed a support vector machine model based on battery charging data for online estimation of battery health status. By comparing partial charging curves, the algorithm can obtain low errors^19^. Gou et al. proposed a new hybrid ensemble data-driven approach by designing a nonlinear autoregressive structure to reduce the remaining useful life (RUL) prediction error of each learning model. A bootstrap-based uncertainty management method is designed to quantitatively evaluate the prediction interval of the RUL to accurately predict the health state of Li-ion batteries^20^.

Two issues need to be considered in aerospace relay health state assessment models. First, assessing the health of aerospace relays is a challenging task due to the high degree of uncertainty caused by various complex factors. The assessment of the relay health state involves inherent uncertainties stemming from several key factors. First, the operating conditions and environmental factors, such as temperature and humidity, can vary and introduce uncertainties in relay performance. Second, the degradation patterns of relays may exhibit non-linear and complex behavior, making it challenging to accurately capture their health states. Third, measurement errors and noise in the acquired data further contribute to the uncertainty in health state assessment. Lastly, the limited availability and quality of historical data for relays can also hinder the accuracy of assessments, as it may not fully capture the diverse operating conditions and failure modes. Therefore, the model must be able to handle this uncertainty and ensure its accuracy. In addition, since aerospace relays are critical components of aircraft system, it is critical that the models constructed are both reliable and easy for users to understand and use. Therefore, it is critical to develop a model that strikes a balance between accuracy and interpretability^6,7,21^.

Belief rule base (BRB) can handle incomplete or uncertain data and provide inference in the face of uncertainty^22,23^. This is because BRB can take into account the belief level associated with each rule, allowing for more nuanced and accurate predictions. Second, BRB is based on IF–THEN explicit semantic modeling and uses evidential reasoning (ER) methods as a transparent inference engine, meaning it allows professionals to understand how a particular prediction was made. This is particularly important in industries such as aerospace, where reliability and transparency are key factors. By understanding how a model arrives at a particular decision, experts can verify the accuracy of the prediction and make any necessary adjustments.

Currently, many scholars have explored various methods to assess the health state of critical systems using BRB. Cheng et al. proposed the multidiscounted BRB (MBRB) to assess the health of large-scale complex electromechanical systems. MBRB model considers expert reliability discount to reduce the negative impact of cognitive uncertainty on system assessment. In addition, monitoring and environmental uncertainties are also considered. Finally, it shows superior performance in the health assessment of high-speed trains^24^. Zhang et al. proposed a BRB with sliding time window considering correlation and redundancy of input information (BRB-WCR). The method addresses the issues of correlation of characteristics and the high real-time requirements of the system. Finally, the effectiveness of the method was verified using an inertial navigation platform^25^. Zhao et al. developed a method based on BRB with ER to achieve inconsistent fusion and rule-based evaluation. Finally, the health of the lithium-ion battery of the satellite in orbit was assessed using telemetry data and the validity of the method was verified^26^. In a previous study, Zhou et al. performed a health state assessment of aircraft relays based on BRB. Two key features were collected to train the rule base parameters, the reliability of the input features of the BRB in a complex interference environment was considered, and the concept of attribute reliability was proposed to introduce the inference calculation. It is noteworthy that some of the trained parameters deviate strongly from the initial settings of the experts, and some of the rules show incomprehensible distributions of the belief degree^6^.

The BRB-based model for assessing the health state of aerospace relays faces three problems. First, the interpretability of the BRB is likely to be destroyed in the process as the study progresses^6^. Therefore, the interpretability criteria of BRB proposed by Cao et al. need to be considered^7,27^. Second, based on these criteria, designing a set of interpretability maintenance strategies and adapting the related algorithms are urgent issues. How to build a complete evaluation model to achieve high accuracy and interpretability is the last issue to be considered. To address these issues, an interpretable BRB with step-length convergence strategy (IBRB-Sc) evaluation model is proposed in this paper.

The main contributions of this paper include:

1. A new interpretable BRB model is proposed to evaluate the health state of aerospace relays.
2. The interpretable model building is regulated by interpretable criteria.
3. Four interpretable strategies are designed to maintain the interpretability of the model, including the determination of activation rules, parameter optimization constraints, rationalization of the belief distribution, and interpretable step-length convergence strategy.

The rest of the paper is organized as follows: The problem formulation and the construction of the IBRB-Sc model are described in "Problem formulation and construction of the model". The interpretability of IBRB-Sc is considered in "Interpretability of the health state assessment model based on IBRB-Sc". The inference and optimization process of the IBRB-Sc model is described in "Inference and optimization process of the IBRB-Sc model". To demonstrate the validity of the constructed IBRB-Sc model, a case study is examined in "Case study". Finally, the paper is summarized in "Conclusion".

## Problem formulation and construction of the model

The problem formulation is described in "Problem formulation". The IBRB-Sc model for aerospace relay health state assessment is constructed in "Construction of the IBRB-Sc model for aerospace relay health state assessment".

### Problem formulation

When developing health state assessment models with BRB, three key issues must be considered:

#### Problem I

How to ensure the interpretability of the model.

Maintaining the interpretability of health state assessment models is critical^28^. First, interpretability allows professionals to appreciate the evaluation process of the model and evaluate its validity. Second, if a model is not transparent or easily explainable, it may be met with skepticism or mistrust. Third, health state assessment models that are not transparent or interpretable may not meet certain rules. Therefore, this paper considers the general interpretability criteria of BRB.

1 $$ C = \left\{ {C_{1} ,C_{2} , \ldots ,C_{n} } \right\}, $$

where C is the set of interpretable criteria. *n* is the number of proposed interpretable criteria.

#### Problem II

How to design interpretable optimization strategies for optimization algorithm problems.

The optimization-seeking nature of BRB optimization can cause the parameter set to lose its physical meaning, leading to optimization results that are difficult for professionals to understand and accept^27,29^. Additionally, the convergence of the step length in BRB optimization is opaque, which further complicates the interpretability and reliability of the optimization results. Therefore, it is crucial to consider innovative optimization strategies that embed expert knowledge into the algorithm to improve transparency and interpretability.

2 $$ S = \left\{ {C\parallel S_{1} ,S_{2} , \ldots ,S_{m} } \right\}, $$

where *S* is the set of interpretable optimization strategies based on interpretable criteria. *m* is the number of interpretable strategies proposed.

#### Problem III

How to construct a health state assessment model for the aerospace relay.

Aerospace relays play a very important role as the component that connects DC and AC power and converts DC to AC power on the aircraft. If a relay fails, the safety of the aircraft can be seriously compromised. Therefore, it is necessary to build a model that is both accurate and interpretable for health status assessment^22^.

First, how to model initially based on observed attributes with expert knowledge.

3 $$ \varphi \left( {X_{1} ,X_{2} , \ldots ,X_{N} ,K_{\exp ert} } \right) $$

where $\varphi ( \cdot )$ represents the initial modeling process. $X_{1} ,X_{2} , \ldots ,X_{N}$ represents the observed attributes. $K_{{{\text{expert}}}}$ is the expert knowledge.

Second, how the model parameters should be optimized based on the improved optimization algorithm.

4 $$ \Omega_{best} = \Phi \left( {\Omega ,P} \right), $$

where $\Omega$ represents the optimized parameters. $\Omega_{best}$ represents the optimal parameters. $\Phi ( \cdot )$ represents the optimization process. $P$ represents the required algorithm parameters of the optimization process.

Finally, the final evaluation results are obtained based on the model inference.

5 $$ y = f\left( {x,\Omega_{best} } \right), $$

where *x* is the observation data.* y* is the evaluation results. $f( \cdot )$ represents the inference process.

### Construction of the IBRB-Sc model for aerospace relay health state assessment

The BRB is a powerful method for constructing inference systems that can handle uncertain and incomplete information. It is a system based on knowledge and data that can be used to make decisions in complex and uncertain environments^30^.

**Figure 1:**
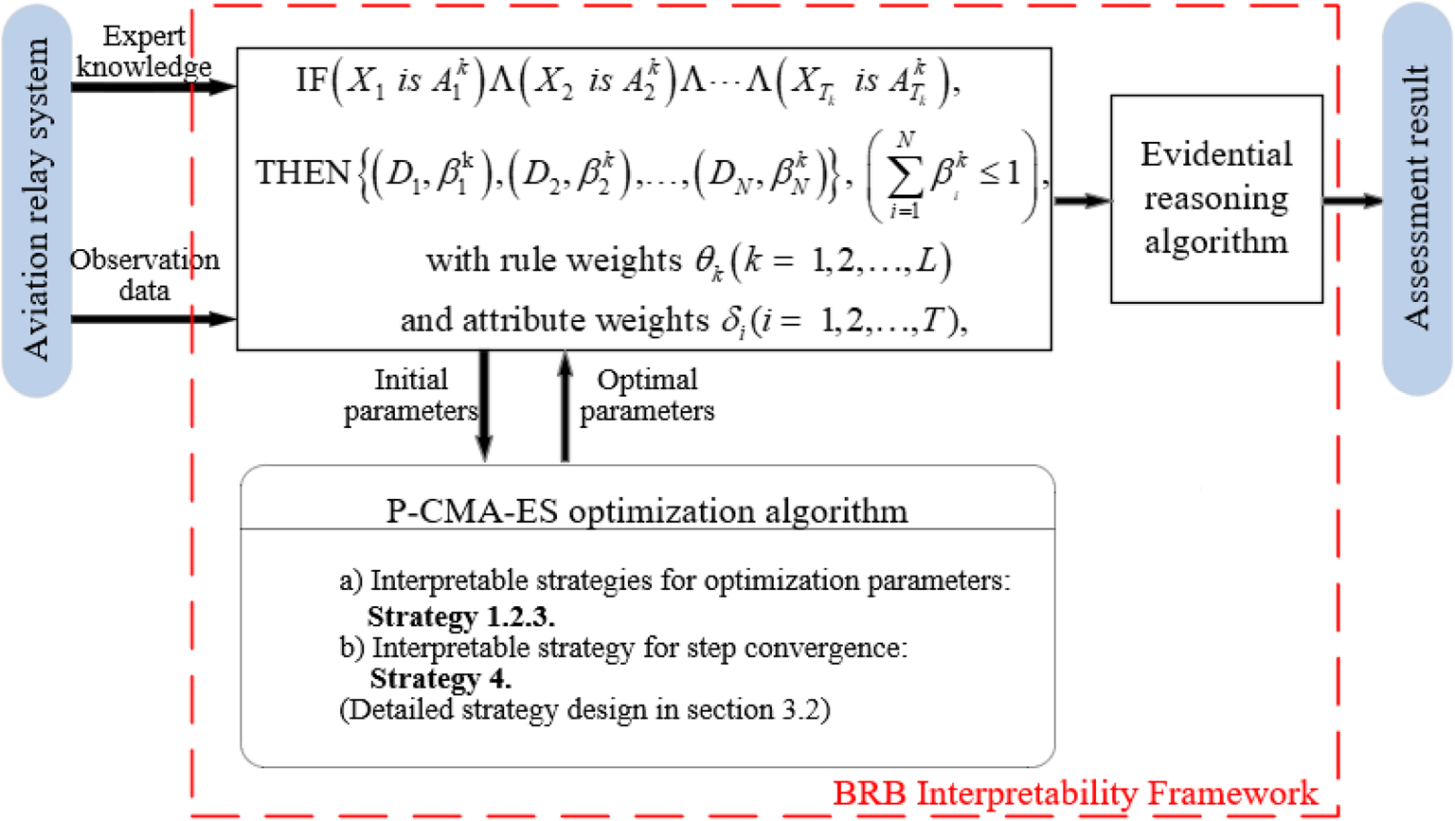
The IBRB-Sc model proposed in this paper for aerospace relay health state assessment.

The BRB is easy to understand based on the IF–THEN rule, and one of the belief rules is constructed as follows:

6 $$ \begin{aligned} & {\text{IF}}\left( {X_{1} \, is \, A_{1}^{k} } \right)\Lambda \left( {X_{2} \, is \, A_{2}^{k} } \right)\Lambda \cdots \Lambda \left( {X_{{T_{k} }} \, is \, A_{{T_{k} }}^{k} } \right), \\ & {\text{THEN}}\left\{ {\left( {D_{1} ,\beta_{1}^{{\text{k}}} } \right),\left( {D_{2} ,\beta_{2}^{k} } \right), \ldots ,\left( {D_{N} ,\beta_{N}^{k} } \right)} \right\}{, }\left( {\sum\limits_{i = 1}^{N} {\beta_{{_{i} }}^{k} \le 1} } \right), \\ & {\text{with}}\,{\text{rule}}\,{\text{weights }}\theta_{k} \left( {k = \, 1,2, \ldots ,L} \right) \\ & {\text{and}}\,{\text{attribute}}\,{\text{weights }}\delta_{i} (i = \, 1,2, \ldots ,T), \\ \end{aligned} $$

where $X_{1} ,X_{2} , \ldots ,X_{{T_{K} }}$ are the antecedent attributes. $A_{i}^{k} \left( {i = 1,2, \ldots ,T_{k} } \right)$ denotes the referential value of the *i*th antecedent attribute. $\theta_{k}$ is the weight of the *k*th rule. $\delta_{i} \left( {i = 1,2, \ldots ,T} \right)$ denotes the weight of the *i*th attribute. *L* denotes the number of rules and $T$ is the number of antecedent attributes. $\beta_{i}^{k} \left( {i = 1,2, \ldots ,N} \right)$ denotes the belief degree of result *D*. The constructed health state assessment model for aerospace relays is shown in Fig. 1.

## Interpretability of the health state assessment model based on IBRB-Sc

The interpretability criteria followed in this paper are demonstrated in "Interpretability criteria", and interpretability strategies are designed in "Strategies for maintaining model interpretability".

### Interpretability criteria

Interpretability is an important consideration when developing models, especially in areas such as aerospace, where decisions based on these models can have a significant impact on the safety of life as well as property. Following interpretability criteria ensures that the entire decision-making process of a model is transparent, understandable, and trustworthy.

**Figure 2:**
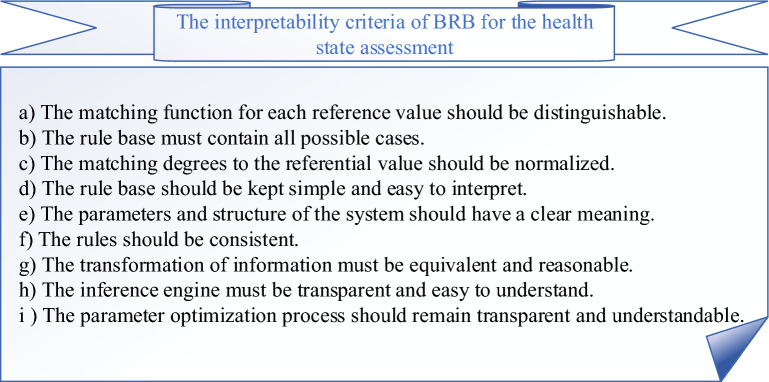
The interpretability criteria of IBRB-Sc for the health state assessment.

The BRB model itself has good interpretability, but as research progresses, an increasing number of deficiencies are identified. Therefore, Cao et al. summarized the interpretability criteria for BRB and guided the direction for subsequent interpretability research^7^. The interpretability criteria of IBRB-Sc for the health state assessment in this paper can be summarized in Fig. 2.

As the proposed interpretability criteria, the interpretability of BRB can be summarized in three aspects.

1. Modeling phase: the rules in BRB consist of IF–THEN statements that can be interpreted and understood by domain experts. Each rule represents a specific condition (IF part) and its corresponding conclusion (THEN part), providing insight into the model inference process. In addition, the specification of the initially set parameters, and improving the completeness and simplicity of the rule base are also important features of the interpretability of the modeling phase.
2. Inference phase: inference mechanisms used in BRB, such as ER algorithms, can be interpreted as understanding how the model combines evidence and makes decisions. The steps involved in the inference process, such as evidence combination, rule activation, and belief updating, can be interpreted and understood.
3. Optimization phase: The interpretability of the optimization phase refers to the extent to which the optimization parameters can be understood and interpreted. Methods that can be used include: adding constraints, increasing expert knowledge, or improving the transparency of the optimization algorithm.

### Strategies for maintaining model interpretability

The use of optimization algorithms greatly improves the accuracy of the model but may also destroy interpretability^7,27^. For BRB, each parameter has its physical meaning and cannot be destroyed. In addition, an interpretable model requires transparency of the process. All these factors limit the application of BRB under some sensitive and complex systems. Therefore, based on the interpretability criteria of BRB proposed in the previous section, several solution strategies are considered in this paper.

*Strategy 1* The corresponding parameters of the inactive rules remain unchanged.

In BRB, the input data will activate the corresponding rules. Then, the next fusion operation is performed to finally obtain the evaluation results^27^. However, in a complex system, it is possible that the collected data do not activate the rules in the whole library. Therefore, during the training of the model, it should be noted that the corresponding parameters of the inactivated rules cannot participate in the optimization. The original optimization algorithm, on the other hand, does not satisfy this point. Therefore, the algorithm needs to be adjusted.

First, the inactivation rules are marked. Second, the original values of the parameters associated with inactive rules are automatically corrected if optimization occurs. The final optimization results improve both the accuracy of the model and make the optimization results acceptable to professionals.

7 $$ \omega_{k} = \left( {w_{1} ,w_{2} , \ldots ,w_{P} } \right), \, k{ = 1,2,} \ldots {,}L, $$

where *p* is the number of input data and $w$ is the activation weight. $\omega_{k}$ is the set of activation weights for the kth rule.

8 $$ m_{k} = \left\{ \begin{gathered} 0,\quad \omega_{k} = 0, \hfill \\ 1,\quad \omega_{k} \ne 0, \hfill \\ \end{gathered} \right. $$

where $m_{k}$ is the labeling factor. If the entire $\omega$ set is 0, this inactive rule is marked.

*Strategy 2* The weight of the optimization is kept in the range of the expert's judgment.

The rule weights and attribute weights represent the initial judgment of the expert for the system. They should not be seriously violated from the initial judgment during the optimization process. Therefore, based on the expert's experience with complex systems, the expert sets a set of optimization ranges and optimizes within the range of the expert's knowledge, making the system significantly more interpretable.

9 $$ \begin{aligned} & \theta_{l} \le \theta_{k} \le \theta_{{\text{u}}} ,\left( {k = 1,2, \ldots ,L} \right) \\ & \delta_{l} \le \delta_{i} \le \delta_{u} ,\left( {i = 1,2, \ldots ,T} \right), \\ \end{aligned} $$

where $\theta_{l}$ and $\delta_{l}$ represent the minimum thresholds for rule weights and attribute weights, respectively. $\theta_{u}$ and $\delta_{u}$ represent the maximum thresholds of the rule weights and attribute weights, respectively.

*Strategy 3* The belief distribution must satisfy the actual system mechanism.

Belief distribution is a feature of BRB, which allows a clearer understanding of the distribution of results^7,8^. However, a random optimization process may undermine logical rationality. For example, as shown in Table 1, the belief level of a student's evaluation as excellent is 0.8, the medium is 0, and the poor is 0.2, which contradicts the realistic logic.

**Table 1:** Belief distribution of a student's evaluation.

Evaluation level	Excellent	Medium	Poor
Belief degree	0.8	0	0.2

Therefore, this paper proposes corresponding strategies to maintain the interpretability of BRB. As shown in Eq. (11), the belief distribution should be convex or progressive.

10 $$ \left( {\beta_{1}^{k} ,\beta_{2}^{k} , \ldots ,\beta_{N}^{k} } \right) \sim S_{3} , $$

11 $$ S_{3} = \left\{ \begin{gathered} \left( {\beta_{1}^{k} \le \beta_{2}^{k} \le \cdots \le \beta_{N}^{k} } \right) \hfill \\ \vee \left( {\beta_{1}^{k} \ge \beta_{2}^{k} \ge \cdots \ge \beta_{N}^{k} } \right) \hfill \\ \vee \left( {\beta_{1}^{k} \le \cdots \le \max \left( {\beta_{2,...,N - 1}^{k} } \right) \ge \cdots \ge \beta_{N}^{k} } \right). \hfill \\ \end{gathered} \right. $$

*Strategy 4* Adopt an interpretable expert-controlled step-length convergence approach.

The opaqueness of step-length convergence can make it difficult to interpret and understand the optimization process, which may lead to a lack of trust in the model. In this paper, the focus is on interpretability, and the step-length convergence of the algorithm needs to consider the controllability and transparency of the convergence process. Therefore, in this paper, an interpretable step-length convergence strategy modulated by experts is designed.

The step-length convergence parameter is given subjectively based on the expert's judgment of the system, with the aim of constraining the optimization range through the expert's confidence in the system's judgment. On the one hand, over-optimization of the parameters can be avoided by setting the initial value of the step length by the expert. On the other hand, the expert can control the rate of convergence of the step length to control the optimization range. For example, if the expert is confident in the provided knowledge, the optimization process focuses on a small search range near the initial parameters. Conversely, if the expert is not confident in the provided knowledge, the step length is kept converging slowly in the early stages to ensure that there is enough time for optimization in a larger range. This interpretable strategy achieves the controllability of step-length convergence by embedding expert judgment, and it ensures that the optimization algorithm is transparent and understandable, which greatly improves the interpretability of the BRB optimization process. The proposed step-length convergence equation is as follows:

12 $$ \varepsilon^{{\left( {{\text{g}} + 1} \right)}} = k_{1} \times \left( {1 - \frac{g}{G}} \right)^{{k_{2} }} + V, $$

where *k*_*1*_ controls the rate of decline of the convergence curve and *k*_*2*_ controls the degree of decline of the convergence curve. The minimum step length threshold *V* guarantees the convergence step length to be minutely sought in the final stage. These three parameters are given by the experts. *g* is the number of iterations. *G* is the set maximum number of iterations.

## Inference and optimization process of the IBRB-Sc model

The inference process of BRB is described in "Inference process of the IBRB-Sc model", and the optimization process of BRB is presented in "Optimization process of the IBRB-Sc model".

### Inference process of the IBRB-Sc model

This paper uses the ER method for reasoning about BRB. The method is transparent to uncertain information and can be used to guide the evaluation, understand the conclusions, and use them as a basis for action^31–33^. The specific inference process is as follows:

*Step 1* First, the input qualitative information or quantitative information is transformed into a belief distribution.

13 $$ S(x_{i} ) = \{ (A_{i,j} ,a_{i,j} ),\quad i = 1,2, \ldots ,M;\,j = 1,2, \ldots ,J_{i} \} , $$

where *A*_*i,j*_ denotes the *j*th referential value of the *i*th input attribute. *a*_*i,j*_ denotes the matching degree of *A*_*i,j*_.

*Step 2* The activation weights are calculated, and the activation rules are determined.

14 $$ w_{k} = \frac{{\theta_{k} \prod\nolimits_{i = 1}^{T} {(\alpha_{i,j}^{k} )^{{^{{\overline{\delta }_{i} }} }} } }}{{\sum\nolimits_{l = 1}^{L} {\theta_{l} } \prod\nolimits_{i = 1}^{T} {(\alpha_{i,j}^{k} )^{{^{{\overline{\delta }_{i} }} }} } }}, $$

15 $$ \overline{\delta }_{i} = \delta_{i} /\mathop {\max }\limits_{{i = 1,2, \ldots ,T_{k} }} \{ \delta_{i} \} . $$

*Step 3* The synthesis of activation rules is performed, and the belief degree of the results is calculated.

16 $$ \beta_{n} = \frac{{\left[ {\prod\nolimits_{k = 1}^{L} {\left( {w_{k} \beta_{n}^{k} + \gamma_{n,i}^{k} } \right) - \prod\nolimits_{k = 1}^{L} {\left( {\gamma_{n,i}^{k} } \right)} } } \right]}}{{\sum\nolimits_{n = 1}^{N} {\prod\nolimits_{k = 1}^{L} {\left( {w_{k} \beta_{n}^{k} + \gamma_{n,i}^{k} } \right) - \left( {N - 1} \right)\prod\nolimits_{k = 1}^{L} {\left( {\gamma_{n,i}^{k} } \right)} } } - \prod\nolimits_{k = 1}^{L} {\left( {1 - w_{k} } \right)} }}, $$

17 $$ \gamma_{n,i}^{k} = 1 - w_{k} \sum\limits_{i = 1}^{N} {\beta_{i}^{k} } , $$

where $\beta_{n}$ represents the belief level of the results. $\gamma$ represents the intermediate parameter.

*Step 4* The belief distribution of the synthesized results is obtained.

18 $$ S\left( {A^{^\circ } } \right) = \left\{ {\left( {D_{n} ,\beta_{n} } \right);\quad n = 1,2, \ldots ,N} \right\}, $$

where $A^{^\circ }$ denotes the input vector.

*Step 5* The effectiveness is calculated, and the final results are obtained.

19 $$ u\left( {S\left( {A^{^\circ } } \right)} \right) = \sum\limits_{n = 1}^{N} {u\left( {D_{n} } \right)} \beta_{n} , $$

where *u*(*D*_*n*_) denotes the utility value.

### Optimization process of the IBRB-Sc model

The projection covariance matrix adaptive evolution strategy (P-CMA-ES) can be very good for model optimization and is widely used, especially for BRB optimization^34–38^. However, if the initial P-CMA-ES is used directly for the optimization of BRB, it will result in an opaque process, uncontrollable behavior, and unintelligible results. Therefore, this paper fully considers the interpretability criteria and designs a series of interpretability strategies to protect the interpretability of BRB. The specific steps of the improved interpretable projection covariance matrix adaptive evolution strategy (IP-CMA-ES) are as follows:

First, a new objective function is constructed.

20 $$ \begin{aligned} & Min\left\{ {\zeta \left( {\beta ,\theta ,\delta } \right)} \right\} \\ & s.t. \, \theta_{l} \le \theta \le \theta_{{\text{u}}} , \\ & \delta_{l} \le \delta \le \delta_{u} , \\ & \sum\limits_{i = 1}^{N} {\beta_{{_{i} }}^{k} \le 1} , \, \beta \sim S_{3} , \\ \end{aligned} $$

where $\zeta$ denotes the mean squared error (MSE).

*Step 1* Set the initial optimization reference value.

Iteration number G, step length $\varepsilon^{0}$, covariance matrix $C^{0}$, population size $\lambda$.

*Step 2* Generate the population.

21 $$ \Omega_{k}^{g + 1} = m^{\left( g \right)} + \varepsilon^{\left( g \right)} N\left( {0,C^{\left( g \right)} } \right),\quad k = 1,2, \ldots ,\lambda , $$

where *N* is the normal distribution. Note that the initial mean value *m* is expert knowledge. $\Omega$ is the generated population and the parameter vector of BRB.* g* is the number of iterations.

*Step 3* Maintaining the interpretability of the optimization process.

Strategy 1: The corresponding parameters of the inactive rules remain unchanged.

22 $$ \left\{ {m_{k} \parallel \left( {\left( {\beta_{1}^{k} ,\beta_{2}^{k} , \ldots ,\beta_{N}^{k} } \right),\theta_{k} } \right)} \right\}. $$

Based on the labeling factor, the parameters of the inactivated rules are not optimized by the algorithm, maintaining the initial expert knowledge.

Strategy 2: The weight of the optimization is kept in the range of the expert's judgment.

23 $$ \begin{aligned} & \theta_{l} \le \theta_{k} \le \theta_{{\text{u}}} ,\left( {k = 1,2, \ldots ,L} \right) \\ & \delta_{l} \le \delta_{i} \le \delta_{u} ,\left( {i = 1,2, \ldots ,T} \right). \\ \end{aligned} $$

Strategy 3. The belief distribution must satisfy the actual system mechanism.

24 $$ \left( {\beta_{1}^{k} ,\beta_{2}^{k} , \ldots ,\beta_{N}^{k} } \right) \sim S_{3} , $$

25 $$ S_{3} = \left\{ \begin{gathered} \left( {\beta_{1}^{k} \le \beta_{2}^{k} \le \cdots \le \beta_{N}^{k} } \right) \hfill \\ \vee \left( {\beta_{1}^{k} \ge \beta_{2}^{k} \ge \cdots \ge \beta_{N}^{k} } \right) \hfill \\ \vee \left( {\beta_{1}^{k} \le \cdots \le \max \left( {\beta_{2,...,N - 1}^{k} } \right) \ge \cdots \ge \beta_{N}^{k} } \right). \hfill \\ \end{gathered} \right. $$

*Step 4* Project the solution into the hyperplane:

26 $$ A_{e} \Omega_{k}^{{\left( {g + 1} \right)}} \left( {1 + n_{e} \times \left( {j - 1} \right):n_{e} \times j} \right) = 1,\quad j = 1,2, \ldots ,N + 1, $$

where* A*_*e*_ denotes the parameter vector. *n*_*e*_ and *j* are the quantities of variables in equality constraints and equality constraints in $\Omega_{k}^{\left( g \right)}$, respectively.

The projection operation:

27 $$ \begin{aligned} & \Omega_{k}^{{\left( {g + 1} \right)}} \left( {1 + n_{e} \times \left( {j - 1} \right):n_{e} \times j} \right) = \Omega_{k}^{{\left( {g + 1} \right)}} \left( {1 + n_{e} \times \left( {j - 1} \right):n_{e} \times j} \right) \\ & \quad - A_{e}^{T} \times \left( {A_{e} \times A_{e}^{T} } \right)^{ - 1} \times \Omega_{k}^{{\left( {g + 1} \right)}} \left( {1 + n_{e} \times \left( {j - 1} \right):n_{e} \times j} \right) \times A_{e} . \\ \end{aligned} $$

*Step 5* Select the optimal solution and update the mean:

Calculate and sort the MSE value:

28 $$ \zeta \left( {\Omega_{1:\lambda }^{{\left( {g + 1} \right)}} } \right) \le \zeta \left( {\Omega_{2:\lambda }^{{\left( {g + 1} \right)}} } \right) \le \cdots \le \zeta \left( {\Omega_{\lambda :\lambda }^{{\left( {g + 1} \right)}} } \right), $$

where $\Omega_{i:\lambda }^{{\left( {g + 1} \right)}}$ is the *i*th solution in $\lambda$ solutions.

The optimal subgroup is updated:

29 $$ m^{{\left( {g + 1} \right)}} = \sum\limits_{i = 1}^{\mu } {\varpi_{i} \Omega_{i:\lambda }^{{\left( {g + 1} \right)}} } , $$

where $\varpi$ is the weight set based on the fitness value. $\mu$ is the number of offspring populations. In the algorithm, the (*g* + 1)th generation population is generated based on the expected local evolution of the partially optimal subpopulation in the *g*th generation, thus finding the better offspring population. Therefore, the direction of population evolution is determined.

*Step 6* Update the covariance matrix of the population:

30 $$ \begin{aligned} C^{{\left( {g + 1} \right)}} & = \left( {1 - c_{1} - c_{2} } \right) \cdot C^{\left( g \right)} + c_{1} p_{c}^{{\left( {g + 1} \right)}} \left( {p_{c}^{{\left( {g + 1} \right)}} } \right)^{T} \\ & \quad + c_{2} \sum\limits_{i = 1}^{\tau } {\varpi_{i} \left( {\frac{{\Omega_{i:\lambda }^{{\left( {g + 1} \right)}} - m^{\left( g \right)} }}{{\varepsilon^{\left( g \right)} }}} \right)} \left( {\frac{{\Omega_{i:\lambda }^{{\left( {g + 1} \right)}} - m^{\left( g \right)} }}{{\varepsilon^{\left( g \right)} }}} \right)^{T} , \\ \end{aligned} $$

31 $$ p_{c}^{{\left( {g + 1} \right)}} = \left( {1 - c_{c} } \right) \cdot p_{c}^{\left( g \right)} + \sqrt {c_{c} \left( {2 - c_{c} } \right)} \cdot \left( {\sum\limits_{i = 1}^{\tau } {\varpi_{i}^{2} } } \right)^{ - 0.5} \cdot \left( {m^{{\left( {g + 1} \right)}} - m^{\left( g \right)} } \right)/\varepsilon^{\left( g \right)} , $$

where *c*_*1*_ and* c*_*2*_ denote the learning rate, *p*_*c*_ is the evolutionary path of the covariance matrix path, and *c*_*c*_ is the backward time horizon of the evolution path.

*Step 7* Update the step length:

32 $$ \varepsilon^{{\left( {{\text{g}} + 1} \right)}} = k_{1} \times \left( {1 - \frac{g}{G}} \right)^{{k_{2} }} + V, $$

where $\varepsilon^{{\left( {{\text{g}} + 1} \right)}}$ represents the iterative step length of the *g* + 1 generation population. In the algorithm, the step length mainly controls the ability of global search and local search of the population.

## Case study

The initial IBRB-Sc constructed with expert knowledge is described in "Construct the initial BRB with expert knowledge". The optimization and testing of IBRB-Sc are described in "Optimization and testing of IBRB-Sc". The interpretable analysis is described in "Interpretability analysis". Finally, a comparative study is performed in "Comparative study".

Aerospace relays play a critical role in the operation of spacecraft, and the reliability of these relays is essential for ensuring mission success. Therefore, it is essential to use models that are transparent, understandable, and trustworthy to assess the health status of these relays. Interpretable models can help domain experts identify potential problems early, leading to faster diagnosis and repair of faulty relays. In addition, interpretable models can increase confidence in the decision-making process, ultimately leading to more effective decisions. Therefore, the use of interpretable models to assess the health status of aerospace relays is critical to ensuring mission success and maintaining the safety of astronauts and space equipment.

### Construct the initial BRB with expert knowledge

**Figure 3:**
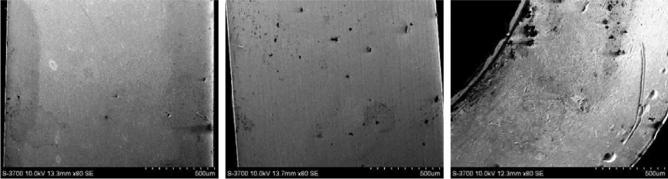
Using scanning electron microscopy, the contact chip was observed to be in three different states of health.

This paper verifies the validity of the IBRB-Sc model through the study of the JRC-7M aerospace relay. Three different states (high health, medium health, and low health) of the contact chip observed by scanning electron microscopy are shown in Fig. 3. The number of failure points increases significantly in low health states, which hurts the operational status of aerospace relays. The results of the study emphasize the importance of accurate and reliable health state assessment models for maintaining the proper operation of aerospace relays.

In the experimental setup, the health states of the relays are given different labels to represent their actual conditions. Data is collected from relays in different health states through a series of carefully designed experiments. This collected data is then analyzed and filtered by experts to give each health state an appropriate label. A value between 0 and 1 is usually used to distinguish and quantify the degree of deterioration of the health condition in the labeled state. This approach allows a clear representation of the health status of the relays, facilitating an effective health assessment and decision-making process.

**Figure 4:**
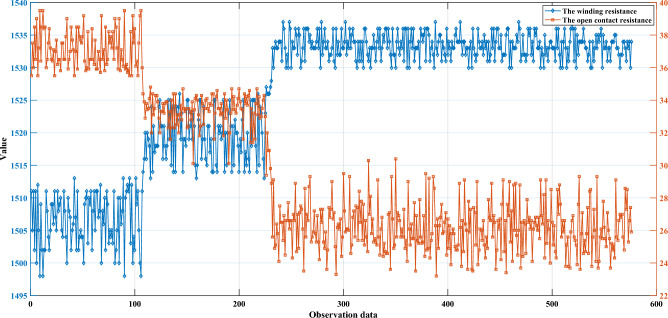
Two observed attributes were collected in three different health states.

In this paper, winding resistance (WR) and open contact resistance (OR) were selected as two attributes in the model^7^, and 576 sets of data in three states were selected. In practice, WR and OR are usually measured periodically during maintenance or test procedures. Intermittent measurements at specific intervals can provide sufficient information to assess their health. These periodic measurements can be used to track changes in resistance over time and to identify any deviations from expected behavior that may indicate potential health problems. To measure WR and OR, the following test experiments were performed on the relay: (1) WR was measured by applying a fixed 4 V voltage to the windings. (2) OR was measured by adding a fixed current to the contacts when a fixed 10 mA voltage was applied to the windings. The observed data in the three health states are shown in Fig. 4. Five reference points are selected for WR and OR, small (S), relatively small (RS), medium (M), relatively large (RL), and large (L), and the corresponding reference values and weights are shown in Table 2. The reference values for the health states of aerospace relays are listed in Table 3. The relevant parameters of the initial rule are shown in Table A1. The settings of these parameters are determined by experts, which include reference values and reference intervals. Under the premise of this study, it is established that expert knowledge, which serves as a fundamental pillar of the research, is presumed to be inherently reliable and trustworthy. The belief rule for the health state assessment model based on IBRB-Sc is expressed as follows:

33 $$ \begin{aligned} & {\text{IF}}\left( {WR \, is \, A_{1}^{k} } \right)\Lambda \left( {OR \, is \, A_{2}^{k} } \right), \\ & {\text{THEN}}\left\{ {\left( {H,\beta_{1}^{{\text{k}}} } \right),\left( {M,\beta_{2}^{k} } \right),\left( {L,\beta_{3}^{k} } \right)} \right\}{, }\left( {\sum\limits_{i = 1}^{3} {\beta_{{_{i} }}^{k} \le 1} } \right), \\ & {\text{with}}\,{\text{rule}}\,{\text{weights }}\theta_{k} \left( {k = \, 1,2, \ldots ,25} \right) \\ & {\text{and}}\,{\text{attribute}}\,{\text{weights }}\delta_{i} (i = \, 1,2), \\ \end{aligned} $$

**Table 2:** The initial weight of attributes and reference values.

Attribute	Attribute weight	Attribute weight constraint	Referential values				
	$\delta_{i}$	$\delta_{i} \sim S_{2}$	S	RS	M	RL	L
Winding resistance	1	0.6 ~ 1	1497.5	1511	1519	1530	1537.5
Open contact resistance	1	0.6 ~ 1	23.1	28.2	33.3	35.8	39.6

**Table 3:** Health states of aerospace relay.

Health states	H	M	L
Referential value	1	0.5	0

### Optimization and testing of IBRB-Sc

In the model training part, two-thirds of the data are randomly selected. The remaining one-third of the data are used as the test. The experiments were conducted in Windows 10 development environment and used Matlab software version R2016a. For this study, no additional external libraries or frameworks were used to ensure focus and control of the experimental setup. The optimization parameters are set as shown in Table 4.

**Table 4:** Optimization parameters of the model.

The parameter	Value	The parameter	Value
*G*_*max*_	200	$\varepsilon^{0}$	0.2
$\lambda$	23	*k*_*1*_	0.2
*k*_*2*_	2.95	*V*	1E−05

**Figure 5:**
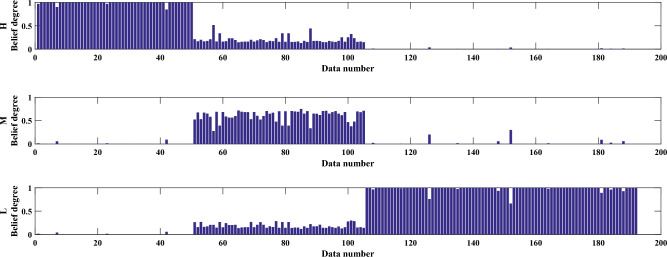
The belief distribution of the health state assessment results of the JRC-7M aerospace relay.

**Figure 6:**
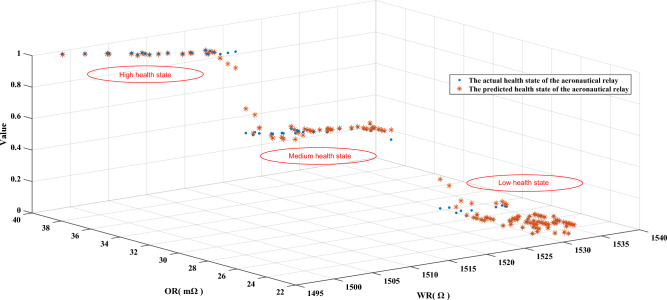
The final health state assessment results of the JRC-7M aerospace relay.

The results of the study revealed that the optimized evaluation model can effectively estimate the health status of aerospace relays. The optimized rules are shown in Table A2. The MSE of the optimized evaluation model was found to be 6.918E−04, which indicates that the model's accuracy is high. The paper conducted 20 experiments to test the robustness of the model and found that the mean and variance of the MSE were 7.425E−04 and 1.001E−08, respectively. The small variance value of the MSE implies that the constructed health state evaluation model has strong robustness and can provide reliable health state assessments of aerospace relays. The belief distribution of the results and the final evaluation results are shown in Figs. 5 and 6, respectively.

### Interpretability analysis

To demonstrate the effectiveness of the proposed strategy, this section provides a detailed validation analysis for the interpretable model.

**Figure 7:**
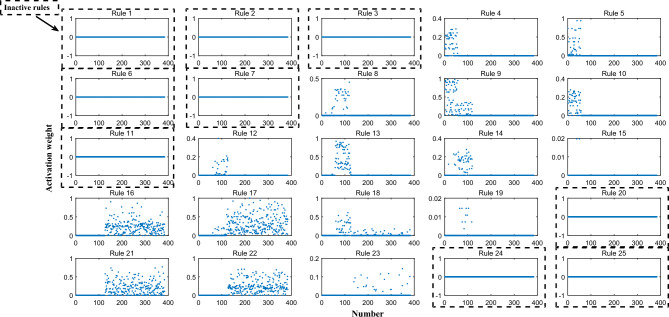
The situation where rules in the rule base are activated by the data entered.

**Figure 8:**
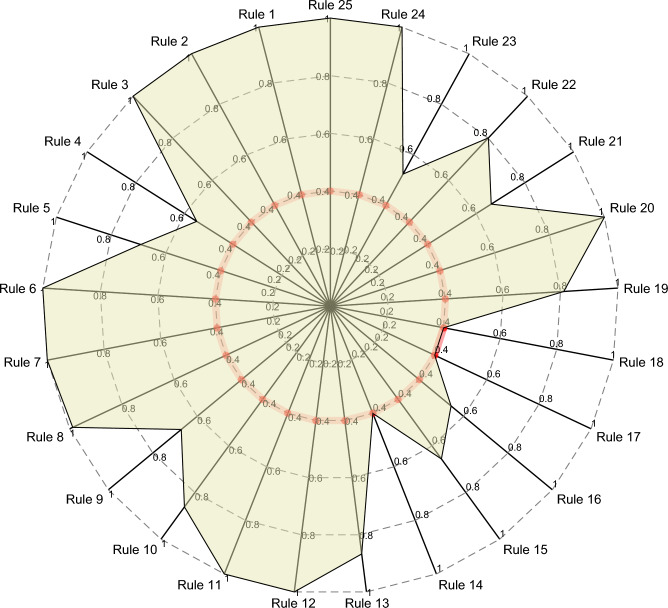
Weights of the optimized rules, where the red line is the constraint setting.

**Figure 9:**
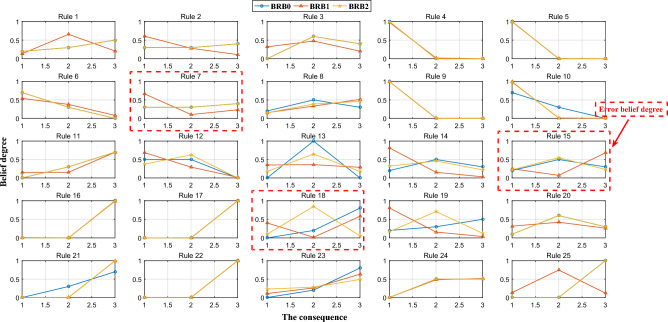
Comparison of the belief distributions of the 25 rules for BRB0, BRB1, and BRB2.

- The proposed strategy protects the original parameters of the unactivated weights, which increases the experts' trust in the model. In Fig. 7, 16 rules are activated, while 9 rules are not activated. As shown in Table A2, the proposed strategy successfully preserves the interpretability of the model and prevents it from experiencing over optimization that is not understood by professionals. This ensures the reliability of the optimization results.

b. Figure 8 represents the optimized rule weights, which clearly shows the importance of the rules in the actual system. The red line in the figure indicates the optimization threshold set by the expert, and the optimization process is carried out within the interval given by the expert. In addition, it can be seen that the marked inactivated rule factors protect the original values of the parameters. Thus, the proposed strategy greatly improves the interpretability of the model.

c. Figure 9 shows the belief distribution for each rule in the BRB rule base. The optimization process adjusts the distributions, resulting in improved accuracy of the results. However, there are some problems, as seen in the red boxed line in the figure, where the belief distribution of BRB1 clearly contradicts the actual system. BRB2, on the other hand, maintains the correctness of the belief distribution. In addition, the corresponding belief degree of the inactivated rules retains the expert knowledge, which fully ensures the interpretability of the model and increases trust from experts in the model.

d. In optimization algorithms, step-length convergence plays a key role in the search ability of the algorithm. In this study, the main concern is interpretability, which requires a controllable and transparent step-length convergence strategy. In this regard, an interpretable step-length convergence strategy incorporating expert knowledge is proposed.

**Figure 10:**
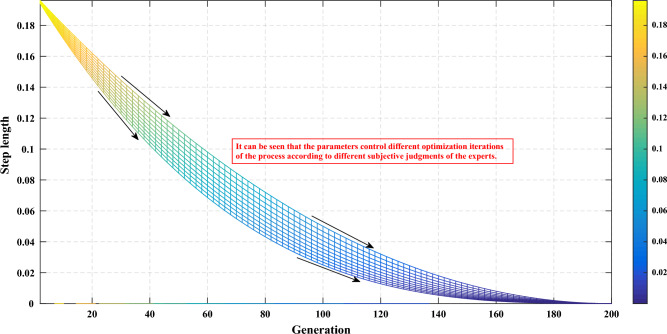
Different step-length convergence processes according to different subjective judgments of experts.

Experts determine step-length convergence parameters based on their judgment of the system. These parameter values are subjective and reflect the expert's confidence in the knowledge provided. As shown in Fig. 10, if the expert's confidence is high, the algorithm emphasizes small exact optimization searches. Conversely, if the expert's confidence is low, the algorithm maintains a slower iteration rate to perform a broader optimization search. This interpretable strategy introduces controllability to the step-length convergence process by incorporating the expert's judgment.

**Figure 11:**
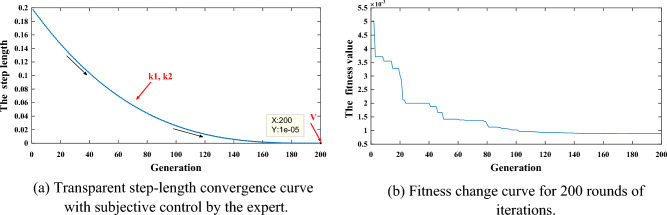
Step-length convergence and the fitness change curves in this experiment.

The step length convergence and fitness optimization process for this experiment is shown in Fig. 11. It can be seen that in the early convergence phase, the algorithm needs to ensure that it can search a large range of the solution space, and hence there are large jumps. In the late convergence phase, the algorithm performs a slow but accurate search for the optimal solution, further searching for the optimal solution within the solution space determined in the early phase. In addition, the proposed strategy ensures the transparency and understandability of the optimization algorithm. This greatly improves the credibility and applicability of the BRB model and promotes its wide application in the real world.

### Comparative study

For ease of exposition, the BRB using the initial expert knowledge is denoted by BRB0, the BRB using the original optimization algorithm is denoted by BRB1, and the IBRB-Sc model is denoted by BRB2.

- Comparison of BRB0, BRB1, and BRB2.

**Figure 12:**
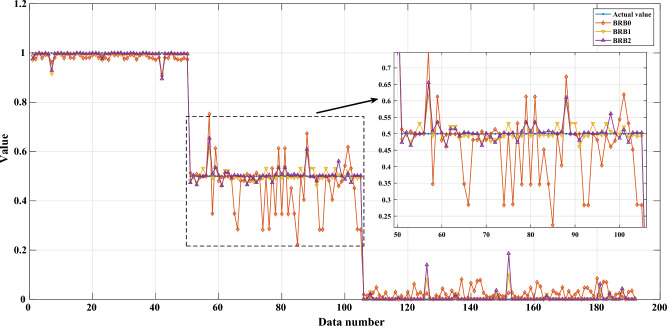
Comparison of assessment accuracy using MSE values for BRB0, BRB1, and BRB2.

As shown in Fig. 12, the BRB0 is accurate for the high health state and low health state, but in the medium health state, the expert's judgment results fluctuate more due to the complex system mechanism or the complicated environment. BRB1 and BRB2, after optimization, greatly improve the evaluation accuracy. BRB1 is more accurate than BRB2. The reason is that BRB1 does not consider interpretable protection when using the accuracy-oriented optimization algorithm. BRB2, on the other hand, achieves good results in terms of accuracy and interpretability.

b. Comparison of BRB1 and BRB2 with Artificial Neural Network (ANN), K-Nearest Neighbors (KNN), and Radial Basis Function (RBF).

In this paper, comparison experiments were conducted for 10 rounds using the same training set and test set. As shown in Table 5, BRB1, RBF, and ANN have higher accuracy than BRB2. KNN has higher MSE values due to prediction errors at several points, but the rest are accurately evaluated. Although the accuracy of BRB2 is slightly lower than that of the other models, BRB2 offers several advantages over black box models in terms of interpretability. BRB2 uses a set of transparent rules that can be easily understood by domain experts, allowing them to verify the reasoning process and identify the important features that contribute to the model's output. The belief values assigned to each rule in BRB2 can also provide insight into the contribution of each rule to the model's overall decision. In contrast, data-based models can be difficult to interpret, making it challenging to understand the reasoning behind their output and making them less transparent to domain experts.

**Table 5:** Accuracy comparison of ANN, KNN, RBF, and BRB2.

Model	BRB1	BRB2	ANN	KNN	RBF
Average MSE	4.25E−04	6.76E−04	5.98E−04	3.9E−03	5.21E−04

## Conclusion

This paper presents a new IBRB-Sc model for the health state assessment of aerospace relays. In this paper, interpretability strategies are designed through interpretability criteria. The interpretability of the parameters is fully protected, and a new step-length convergence strategy is designed to balance the global and local search ability of the optimization algorithm. Then, the optimization algorithm is updated to make the interpretability of BRB more satisfying. Experimental results show that the proposed IBRB-Sc model can accurately estimate the health status of aerospace relays with strong robustness.

In conclusion, the proposed IBRB-Sc model provides a powerful tool for the health state assessment of aerospace relays, with improved interpretability and reliability. However, the current expert knowledge is considered reliable, and the reliability of the expert knowledge should be fully considered in the optimization method. Second, in the interpretable step-length convergence method proposed in this paper, the corresponding parameter values are given by the subjective judgment of the experts, and more objective information should be considered in the subsequent research to determine the parameter settings. In addition, the traceability of model inference results is also an important research direction.

### Supplementary Information

Supplementary Information.

## Data Availability

The datasets generated and/or analysed during the current study are not publicly available due Laboratory requirements but are available from the corresponding author on reasonable request.
