# Supplementary material for: A new interpretable belief rule base model with step-length convergence strategy for aerospace relay health state assessment
Source: Sci Rep. 2023 Aug 28;13:14066. doi: 10.1038/s41598-023-41305-z (PMC10462728; doi:10.1038/s41598-023-41305-z)
Supplement: Supplementary file 1 — Supplementary Information. [file 41598_2023_41305_MOESM1_ESM.docx]

# Appendix Table A

Table A1 The initial rules

| NO. | $\text{WR}\wedge\text{OR}$ | The rule weight constraint | Rule weight | The initial belief distribution |
| --- | --- | --- | --- | --- |
|  |  | $\theta_{k}\sim\text{S}_{1}$ | $\theta_{k}$ | $\left\{\beta_{1}^{k},\beta_{2}^{k},\beta_{3}^{k}\right\}$ |
| 1 | S$\wedge$S | 0.4~1 | 1 | { 0.2, 0.3, 0.5 } |
| 2 | S$\wedge$RS | 0.4~1 | 1 | { 0.3, 0.3, 0.4 } |
| 3 | S$\wedge$M | 0.4~1 | 1 | { 0, 0.6, 0.4 } |
| 4 | S$\wedge$RL | 0.4~1 | 1 | { 1, 0, 0 } |
| 5 | S$\wedge$L | 0.4~1 | 1 | { 1, 0, 0 } |
| 6 | RS$\wedge$S | 0.4~1 | 1 | { 0.7, 0.3, 0 } |
| 7 | RS$\wedge$RS | 0.4~1 | 1 | { 0.3, 0.3, 0.4 } |
| 8 | RS$\wedge$M | 0.4~1 | 1 | { 0.2, 0.5, 0.3 } |
| 9 | RS$\wedge$RL | 0.4~1 | 1 | { 1, 0, 0 } |
| 10 | RS$\wedge$L | 0.4~1 | 1 | { 0.7, 0.3, 0 } |
| 11 | M$\wedge$S | 0.4~1 | 1 | { 0, 0.3, 0.7 } |
| 12 | M$\wedge$RS | 0.4~1 | 1 | { 0.5, 0.5, 0 } |
| 13 | M$\wedge$M | 0.4~1 | 1 | { 0, 1, 0 } |
| 14 | M$\wedge$RL | 0.4~1 | 1 | { 0.2, 0.5, 0.3 } |
| 15 | M$\wedge$L | 0.4~1 | 1 | { 0.2, 0.5, 0.3 } |
| 16 | RL$\wedge$S | 0.4~1 | 1 | { 0, 0, 1 } |
| 17 | RL$\wedge$RS | 0.4~1 | 1 | { 0, 0, 1 } |
| 18 | RL$\wedge$M | 0.4~1 | 1 | { 0, 0.2, 0.8 } |
| 19 | RL$\wedge$RL | 0.4~1 | 1 | { 0.2, 0.3, 0.5 } |
| 20 | RL$\wedge$L | 0.4~1 | 1 | { 0.1, 0.6, 0.3 } |
| 21 | L$\wedge$S | 0.4~1 | 1 | { 0, 0.3, 0.7 } |
| 22 | L$\wedge$RS | 0.4~1 | 1 | { 0, 0, 1 } |
| 23 | L$\wedge$M | 0.4~1 | 1 | { 0, 0.2, 0.8 } |
| 24 | L$\wedge$RL | 0.4~1 | 1 | { 0, 0.5, 0.5 } |
| 25 | L$\wedge$L | 0.4~1 | 1 | { 0, 0, 1 } |

Table A2 The optimized rules

| NO. | $\text{WR}\wedge\text{OR}$ | The rule weight constraint | Rule weight | The optimized belief distribution |
| --- | --- | --- | --- | --- |
|  |  | $\theta_{k}\sim\text{S}_{1}$ | $\theta_{k}$ | $\left\{\beta_{1}^{k},\beta_{2}^{k},\beta_{3}^{k}\right\}$ |
| 1 | S$\wedge$S | 0.4~1 | 1 | { 0.2, 0.3, 0.5 } |
| 2 | S$\wedge$RS | 0.4~1 | 1 | { 0.3, 0.3, 0.4 } |
| 3 | S$\wedge$M | 0.4~1 | 1 | { 0, 0.6, 0.4 } |
| 4 | S$\wedge$RL | 0.4~1 | 0.549 | { 0.999, 0.001, 0 } |
| 5 | S$\wedge$L | 0.4~1 | 0.692 | { 0.996, 0.003, 0.001 } |
| 6 | RS$\wedge$S | 0.4~1 | 1 | { 0.7, 0.3, 0 } |
| 7 | RS$\wedge$RS | 0.4~1 | 1 | { 0.3, 0.3, 0.4 } |
| 8 | RS$\wedge$M | 0.4~1 | 0.988 | { 0.151, 0.388, 0.461 } |
| 9 | RS$\wedge$RL | 0.4~1 | 0.672 | { 1, 0, 0 } |
| 10 | RS$\wedge$L | 0.4~1 | 0.862 | { 0.978, 0.017, 0.005 } |
| 11 | M$\wedge$S | 0.4~1 | 1 | { 0, 0.3, 0.7 } |
| 12 | M$\wedge$RS | 0.4~1 | 0.999 | { 0.374, 0.626, 0 } |
| 13 | M$\wedge$M | 0.4~1 | 0.867 | { 0.176, 0.648, 0.176 } |
| 14 | M$\wedge$RL | 0.4~1 | 0.4 | { 0.321, 0.46, 0.219 } |
| 15 | M$\wedge$L | 0.4~1 | 0.655 | { 0.221, 0.55, 0.229 } |
| 16 | RL$\wedge$S | 0.4~1 | 0.544 | { 0, 0.002, 0.998 } |
| 17 | RL$\wedge$RS | 0.4~1 | 0.4 | { 0, 0, 1 } |
| 18 | RL$\wedge$M | 0.4~1 | 0.4 | { 0.099, 0.846, 0.055 } |
| 19 | RL$\wedge$RL | 0.4~1 | 0.812 | { 0.169, 0.709, 0.122 } |
| 20 | RL$\wedge$L | 0.4~1 | 1 | { 0.1, 0.6, 0.3 } |
| 21 | L$\wedge$S | 0.4~1 | 0.661 | { 0.001, 0.004, 0.995 } |
| 22 | L$\wedge$RS | 0.4~1 | 0.801 | { 0, 0, 1 } |
| 23 | L$\wedge$M | 0.4~1 | 0.523 | { 0.23, 0.281, 0.489 } |
| 24 | L$\wedge$RL | 0.4~1 | 1 | { 0, 0.5, 0.5 } |
| 25 | L$\wedge$L | 0.4~1 | 1 | { 0, 0, 1 } |
